# RNA sequencing and bioinformatics analysis of human lens epithelial cells in age-related cataract

**DOI:** 10.1186/s12886-021-01915-5

**Published:** 2021-03-26

**Authors:** Zhongying Wang, Dongmei Su, Shanhe Liu, Guiqian Zheng, Gaobo Zhang, Tingsong Cui, Xu Ma, Zhaoyi Sun, Shanshan Hu

**Affiliations:** 1 Department of Ophthalmology, Hongqi Hospital of Mudanjiang Medical College, Mudanjiang, 157011 Heilongjiang China; 2 Department of Genetics, Center for Genetics, National Research Institute for Family Planning, Beijing, 100081 China

**Keywords:** Age-related cataract, Differentially expressed genes, Bioinformatics analysis, Pathogenic genes

## Abstract

**Background:**

Age-related cataract (ARC) is the main cause of blindness in older individuals but its specific pathogenic mechanism is unclear. This study aimed to identify differentially expressed genes (DEGs) associated with ARC and to improve our understanding of the disease mechanism.

**Methods:**

Anterior capsule samples of the human lens were collected from ARC patients and healthy controls and used for RNA sequencing to detect DEGs. Identified DEGs underwent bioinformatics analyses, including Gene Ontology (GO) and Kyoto Encyclopedia of Genes and Genomes (KEGG) pathway analyses. Subsequently, reverse transcription quantitative RT-qPCR was used to validate the different expression levels of selected genes.

**Results:**

A total of 698 up-regulated DEGs and 414 down-regulated DEGs were identified in ARC patients compared with controls by transcriptome analysis. Through GO and KEGG bioinformatics analysis, the functions of significantly DEGs and their possible molecular mechanisms were determined. Sequencing results were verified by RT-qPCR as being accurate and reliable.

**Conclusions:**

This study identified several genes associated with ARC, which improves our knowledge of the disease mechanism.

## Background

Age-related cataract (ARC) is a common visual disorder caused by lens opacity, and the main cause of blindness in individuals aged over 50 years [1]. With an increasingly aging society, the incidence of ARC is rising sharply [2]. Currently, surgery is the only effective treatment for ARC, but this is associated with an economic burden for patients and may cause complications [3]. Therefore, alternative effective methods are necessary to prevent and treat the occurrence and development of ARC.

Risk factors such as genetics, oxidative stress, ultraviolet radiation, and drug use were previously shown to be closely related to the pathogenesis of ARC [4]. Of these, changes in the expression of multiple genes under oxidative stress damage seem to be key events [5]. Lens epithelial cells are the only nucleated cells in the lens, and as such contain most of the metabolic, protective, permeable, and other regulatory systems [6]. They communicate with the underlying lens fiber cells and respond to cataract-related damage by altering gene expression [7]. Although several genes associated with the pathogenesis of ARC have been identified, many have not been explored. Therefore, the identification of candidate genes for ARC progression in lens epithelial cells will help further understand the disease mechanism and provide an important basis for reducing disease occurrence and for targeted therapy.

In the present study, we performed RNA sequencing on human lens anterior capsule tissue and compared transcriptome data between ARC patients and healthy controls. Differentially expressed genes (DEGs) were analyzed using bioinformatics such as Gene Ontology (GO) and Kyoto Encyclopedia of Genes and Genomes (KEGG) analyses. The accuracy of RNA sequencing was verified using reverse transcription quantitative RTqPCR for 10 selected DEGs. Several genes were identified as being associated with ARC, which furthers our knowledge of the disease mechanism.

## Methods

### Patient and tissue sample collection

Twelve cases of age-related cataract anterior capsule tissue samples were collected from Hongqi Hospital affiliated to Mudanjiang Medical College from January 2019 to March 2019. All the patients were cortical cataracts, including 6 males and 6 females, aged 55 to 64 years. The diagnostic criteria for ARC are non-congenital cataract, non-posterior cataract, and non-metabolic cataract, no hypertension, no diabetes, no glaucoma, no fundus lesions, no uveitis, no eye trauma, and no intraocular surgery history. Twelve cases of normal anterior capsule tissue samples were collected from corneal donors and patients with lens trauma. The information of the samples was displayed in Table 1. Human anterior lens capsule tissue samples were obtained by an ophthalmologist through a continuous annular capsulorhexis of the lens and randomly divided 4 samples into a group. Tissue samples were immediately frozen in liquid nitrogen and stored at − 80 °C for RNA sequencing. This study followed the principles of the Declaration of Helsinki and all participants signed informed consent.

**Table 1:** The information of our patients and normal samples

Characteristics of the age related cataract patients				Characteristics of the Normal person			
Patient	Age	Eye	Gender	Patient	Age	Eye	Gender
1	55	Right	Male	1	43	Right	Male
2	56	Right	Male	2	44	Left	Male
3	56	Left	Female	3	44	Right	Female
4	58	Left	Male	4	45	Left	Female
5	59	Right	Female	5	45	Right	Male
6	60	Left	Female	6	46	Left	Female
7	61	Left	Male	7	47	Left	Male
8	62	Right	Female	8	47	Right	Male
9	62	Right	Female	9	48	Left	Male
10	62	Left	Male	10	48	Left	Female
11	63	Left	Female	11	49	Right	Female
12	64	Right	Male	12	49	Right	Female

Note: There were 12 patients (12 eyes) with ARC in this study, with an average age of 59.83 ± 3.01 years old. And there were 12 normal samples (12 eyes) in this study, with an average age of 46.25 ± 2.05 years old

### RNA extraction

Since the miRNA isolation kit can isolate total RNA containing small RNAs from cultured cells or tissues for better extraction, we selected the mirVana™ miRNA ISOlation Kit (Ambion-1561) to isolate total RNA from tissue samples according to the manufacturer’s protocol. The RNA quality was determined by OD 260/280 absorbance assessment using a NanoDrop 2000 spectrophotometer (Thermo Fisher Scientific, UK). RNA integrity was evaluated using agarose gel electrophoresis stained with ethidium bromide. All RNA samples were stored at − 80 °C for further application.

### RNA library preparation and transcriptome sequencing

The 12 healthy controls samples were randomly divided into 3 groups, with 4 samples in each group. Similarly, the 12 samples from the ARC patients were also randomly divided into 3 groups of 4 samples per group. Since the sample tissue of the anterior lens capsule we used was very small, and less RNA was extracted from a single sample, we combined the samples for RNA sequencing analysis. The total RNA extracted from each group was about 0.8 μg, and the RIN value was above 7. Therefore, we used a low start volume library construction method (NEBNext® Ultra™ II Directional RNA Library Prep Kit for Illumina) for library construction, which includes mRNA enrichment analysis. Briefly, RNA Library Preparation mainly has the following steps: RNA purification and fragmentation, Synthesize First Strand cDNA, Synthesize Second Strand cDNA, Adenylate 3′Ends, Ligate Adapters, Enrich DNA Fragments. The Agilent bioanalyzer 2100 (Agilent Technologies, Santa Clara, CA) was used to check the size and purity of the library. Sequencing was performed on Illumina HiSeq X Ten to generate 125 bp/150 bp paired-end reads.

### Sequence data analysis

The raw data in the fastq format (raw reads) was first processed using Trimmomatic. Then, clean data (clean reads) were obtained by removing low-quality reads, reads containing adapter, and poly-N from raw data. Q30 content represents the percentage of bases with a Qphred value greater than 30 to the total bases. All subsequent analyses were performed using clean data with high quality. HISAT2 was used to compare the sequence of Clean Reads with the specified reference genome [8]. Fragments Per kb Per Million Reads (FPKM) were calculated to quantify gene expression levels. Differentially expressed genes (DEGs) analysis was performed using the DESeq R package, which based DEGs on the negative binomial distribution. The screening conditions for DEGs were *P* < 0.05 with fold change (FC) > 2.

### GO and KEGG enrichment analysis of DEGs

Gene Ontology (GO) analysis (http://geneontology.org/) was used to categorize the function of the DEGs. And the functional description was mainly divided into biological process, cellular component, and molecular function. Kyoto Encyclopedia of Genes and Genomes (KEGG) analysis [9] (http://www.genome.jp/kegg/) was used to predict the signaling pathways in which these DEGs may be involved, KEGG software from the Kanehisa laboratory was used with prior permiossion. The significantly enriched GO and KEGG terms met the criterion of corrected *P* < 0.05.

### Quantitative real-time PCR validation

The remaining RNA after transcriptome was further used for validation studies by qPCR. Total RNA was extracted from the anterior capsule of the normal lens and cataract patients for RNA transcriptome sequencing. Detected the concentration of RNA with a NanoDrop spectrophotometer. Agarose gel electrophoresis detected the integrity of RNA. Quantification was carried out through a two-step reaction process of reverse transcription (RT) and PCR. Each RT reaction consisted of a total of 10 μl of 0.5 μg RNA, 2 μl of 5x Transx all-in-one SuperMix for qPCR, and 0.5 μl of gDNA remover. The reaction was performed on GeneAmp® PCR System 9700 (Applied Biosystems, USA) at 42 °C, 5 s, and 85 °C for 15 min. Next, 10 μl of the RT reaction mixture was diluted 10-fold with nuclease-free water and kept at − 20 °C. Use LightCycler®480II Real-Time PCR instrument (Roche, Switzerland) with 1 μl cDNA, 5 μl 2xPerfectStartTM Green qPCR SuperMix, 0.2 μl forward primer, 0.2 μl PCR reverse primer, and 3.6 μl nuclease-free water. The reaction was incubated in a 384-well optical plate (Roche, Switzerland) at 94 °C for 30 s, and then at 94 °C for 5 s and 60 °C for 30 s for 45 cycles. The primer sequence is synthesized according to the mRNA sequence obtained from the NCBI database (Table 2). Each sample was analyzed in triplicate. The 2^-ΔΔCt^ method was used for data quantification [10].

**Table 2:** Primer sequences of the selected DEGs

Gene Symbol	Forward primer	Reverse primer	Product length	Tm
β-ACTIN	CATTCCAAATATGAGATGCGTT	TACACGAAAGCAATGCTATCAC	133	60
HSF4	TGCAGGAGGCAAGAGAAAG	AGAAGGGCACCAGGTAGA	99	60
LAMA4	ACCTCGGGATACTGCACA	ATTTCTCCGCTGACATCCA	126	60
NLRP3	AAAGGAAGTGGACTGCGA	CCAGCAGGTAGTACATGG	83	60
CRYBA2	TCCGGAGAGTCCAGCACTA	GCAGGAAGATTCAGGAACC	81	60
NRCAM	AGGGATGGCGAAGAATGA	CCTCTTTGCACAACTGCATA	87	60
TGIF1	CTGATGATTACCAAGAGGCG	TGATGTGCCCTACGAAGC	85	60
ID11	GGGCAAGAGGAATTACGTG	CCAGGCTGGATGCAGTTA	110	60
ANGPTL4	CTCAGTCACATTGACTGACG	GGACCTACACACAACAGC	83	60
DUSP1	CTGAACTCAGCACATTCGG	GGATCACACACTGAGTCCT	93	60
CAV2	GCCAGGATTGAATACTTGGAC	TGAACAGAACAGTGGTGCT	93	60

### Statistical analysis

The statistical significance of the experimental data was conducted by a Student’s t-test. The acceptable significance was set as *p < 0.05; **p < 0.01.

## Results

### Descriptive analysis of sequencing data quality assessment

**Fig. 1:**
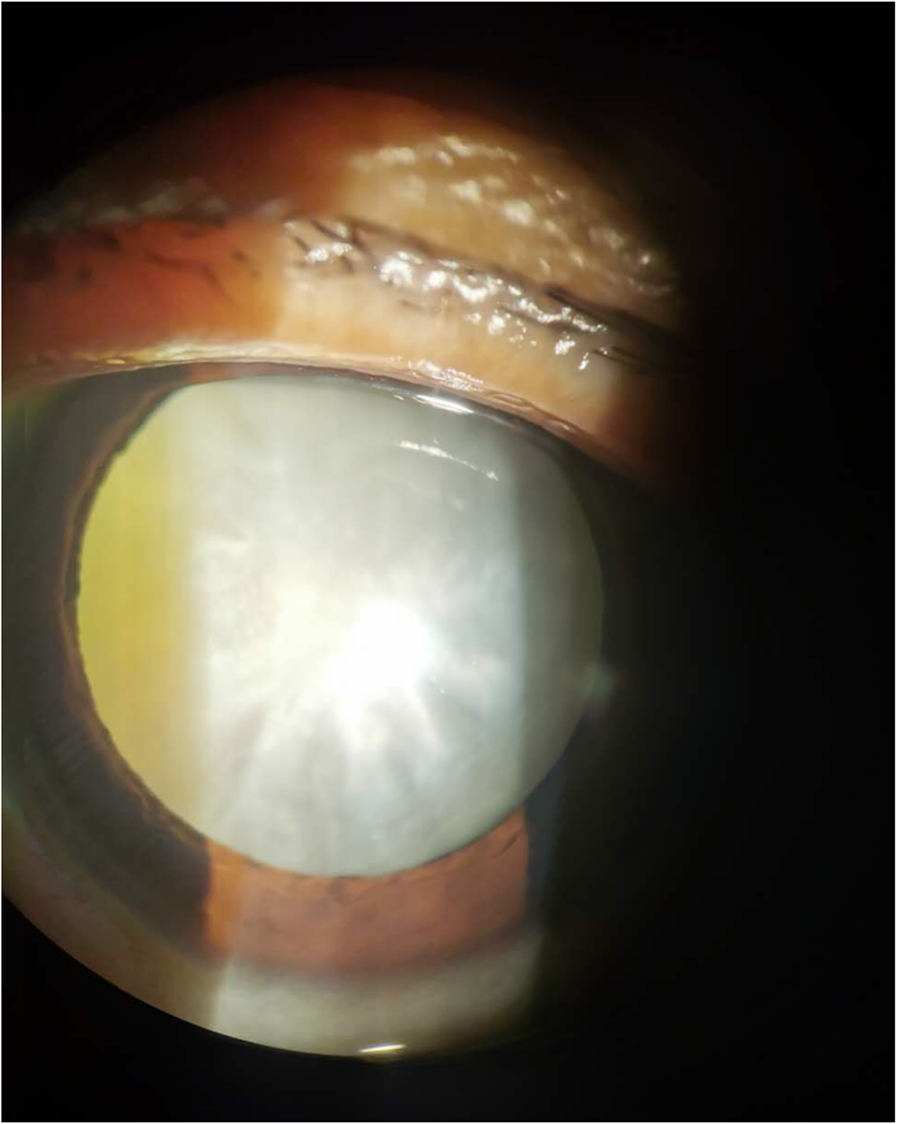
Representative slit-lamp image of the lens with cortical opacity of cataract. From the representative patient, the slit lamp biomicroscopy examination showed the cortical cataract with age. Because the normal subjects with transparent lens were enrolled from corneal transplant donors and ocular trauma patients with lens detachment, a picture of normal control cannot be obtained

Anterior capsule tissue samples were collected from ARC patients (Disease Group, JBZ) and healthy controls (Control group, ZCR). Figure 1 shows an example of a cortical cataract visible as a cloudy lens cortex in a patient with ARC.

We used the Illumina platform to obtain paired-end sequencing data. Because data error can have a major impact on the results, we employed Trimmomatic software to perform quality preprocessing of the original data, and statistically summarize the number of reads throughout the quality control process (Table 3). A total of 55.59 million raw reads were generated from the JBZ1 group, 48.28 million from JBZ2, 48.25 million from JBZ3, 58.89 million from ZCR1, 47.17 million from ZCR2, and 47.97 million from ZCR3. A total of 52.73, 44.64, and 44.59 million clean reads were obtained for JBZ1–3, respectively, and 55.68, 42.94, and 42.95 million for ZCR1–3, respectively. Clean reads ratios were more than 89% for both patients and controls, and over 77% of clean reads could be mapped to human genome sequences. The highest proportion of sequences with only one alignment position on the reference sequence was 89.10% in the JBZ group and 90.47% in the ZCR group. Both JBZ and ZCR groups had approximately 3% multiple alignment positions, and both groups exceeded 90% for Q30 content, representing the percentage of bases with a Qphred value greater than 30. These data indicate that high-quality sequencing was achieved in this study, so subsequent transcriptional analysis could continue.

**Table 3:** Statistics of the sequencing data quality results from all samples

Sample Name	Raw reads	Clean reads	Clean ratio	Mapped reads	Unique mapped	Multi mapped	Q30 (%)
JBZ1	55.59 M	52.73 M	94.86%	92.35%	89.10%	3.25%	94.26%
JBZ2	48.28 M	44.64 M	92.46%	80.42%	77.29%	3.13%	91.21%
JBZ3	48.25 M	44.59 M	92.41%	79.14%	75.74%	3.41%	91.15%
ZCR1	58.89 M	55.68 M	94.55%	93.52%	90.47%	3.05%	94.07%
ZCR2	47.17 M	42.94 M	91.03%	79.03%	75.56%	3.47%	90.42%
ZCR3	47.97 M	42.95 M	89.54%	77.13%	73.34%	3.38%	89.43%

### Analysis of differentially expressed genes (DEGs)

**Fig. 2:**
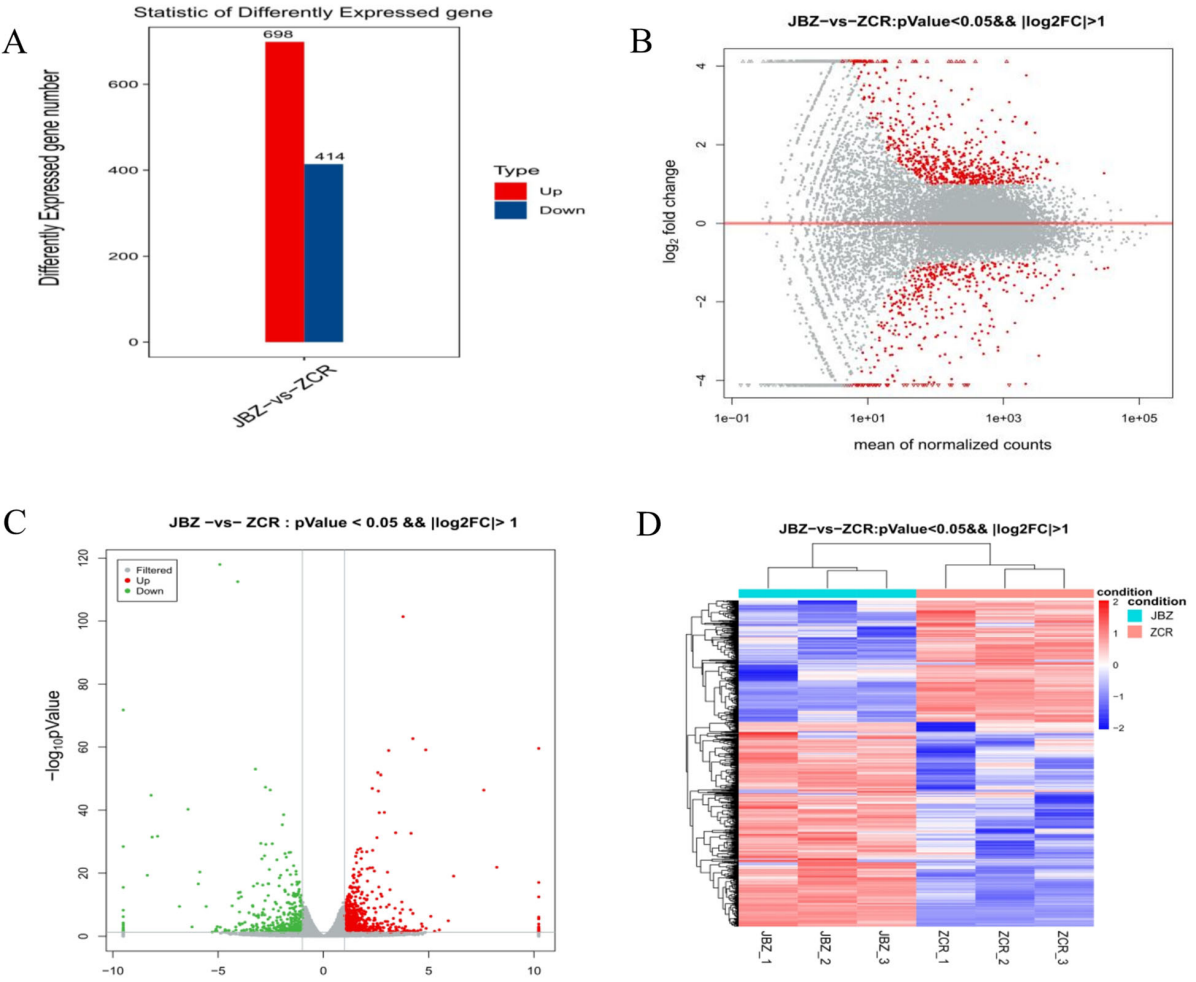
Differentially expressed genes (DEGs) in anterior capsule tissues between the two groups. **a** The number of differentially expressed genes (DEGs) in the JBZ group and ZCR group (P < 0.05, Fold change (FC) > 2). The red histogram represented upregulated gens and a blue histogram represented downregulated gens. **b** The MA plots visualizing the DEGs between the two groups. The X-axis represented the average expression of all samples for comparison after normalization; the Y-axis represented log2FoldChange. The red dots above the red line represented the up-regulated DEGs, and the red dots below the red line represented the down-regulated DEGs. **c** The volcano plot of the DEGs between two groups. Red dots represented significantly up-regulated differential genes, green dots represented significantly down-regulated differential genes, and gray dots represented non-significantly differential genes. **d** Heat-maps of hierarchical clustering analysis of DEGs between two groups. The horizontal ordinate represents the clustering of samples from the JBZ and ZCR groups. The longitudinal coordinates represent the DEGs and the clustering of genes. The red color indicates up-regulated genes, while the blue color indicates down-regulated genes

According to the criteria of *P* < 0.05 and FC > 2, a total of 1112 DEGs were identified between patients and controls. Of these 698 were up-regulated and 414 were down-regulated in the JBZ group compared with the ZCR group (Fig. 2a). Volcano plots, MA plots, and heatmaps of hierarchical clustering showed that the gene expression levels were distinguished (Fig. 2b–d). The top 20 up-regulated genes and top 20 down-regulated genes are listed in Table 4.

**Table 4:** Top 20 upregulated and downregulated differentially expressed genes (DEGs) in the JBZ group compared to the ZCR group

Gene-id	log2FC	Pval	Up-down	Gene-id	log2FC	Pval	Up-down
NR4A2	10.210411523	2.66E-60	Up	DDX3Y	−9.523209533	1.56E-72	Down
NR4A3	8.218022790	1.39E-22	Up	EIF1AY	−8.379329539	4.86E-20	Down
GRIA3	7.601604199	4.05E-47	Up	PVALB	−8.200440025	1.76E-45	Down
ALDH1A2	6.166698445	8.92E-20	Up	CLDN5	−8.150297932	3.56E-32	Down
RGPD1	5.914488712	1.17E-05	Up	RPS4Y1	−7.891783293	1.91E-32	Down
BDNF	5.490073820	0.008935371	Up	LUM	−6.852847001	4.09E-10	Down
UNC5A	5.263108777	0.025651215	Up	C3	−6.447255368	5.17E-41	Down
CAPN8	5.084626101	4.50E-07	Up	SAA1	−6.256915413	0.001039753	Down
SLC16A7	4.861755978	0.002948834	Up	APOD	−5.958632573	2.67E-17	Down
ADAM33	4.843115831	7.86E-60	Up	CHL1	−5.886371002	4.57E-21	Down
MPZ	4.686337999	0.00074464	Up	ABCC3	−5.584424022	4.19E-10	Down
TMEM74B	4.645467019	0.000108655	Up	PRF1	−5.297684334	0.044650321	Down
RRH	4.607044318	0.008796089	Up	KRT78	−5.152554180	0.026495957	Down
NKX3–2	4.331966849	0.029753521	Up	OLAH	−5.139807536	0.017935704	Down
UROC1	4.315361957	0.027729575	Up	FGR	−5.110041644	0.004889969	Down
PRSS55	4.281664428	0.01155766	Up	ISM1	−5.076786718	0.021961678	Down
ATP1A3	4.278127228	0.03309551	Up	GPR160	−5.054228043	0.000798287	Down
SLC7A5	4.231647204	2.05E-63	Up	SERPINA3	−4.933814790	1.01E-118	Down
RGS2	4.230358381	6.09E-08	Up	SGPP2	−4.916332934	0.001338125	Down
ATOH7	4.228904411	0.01977059	Up	FABP9	−4.767243914	0.04901258	Down

### Analysis of GO enrichment

**Fig. 3:**
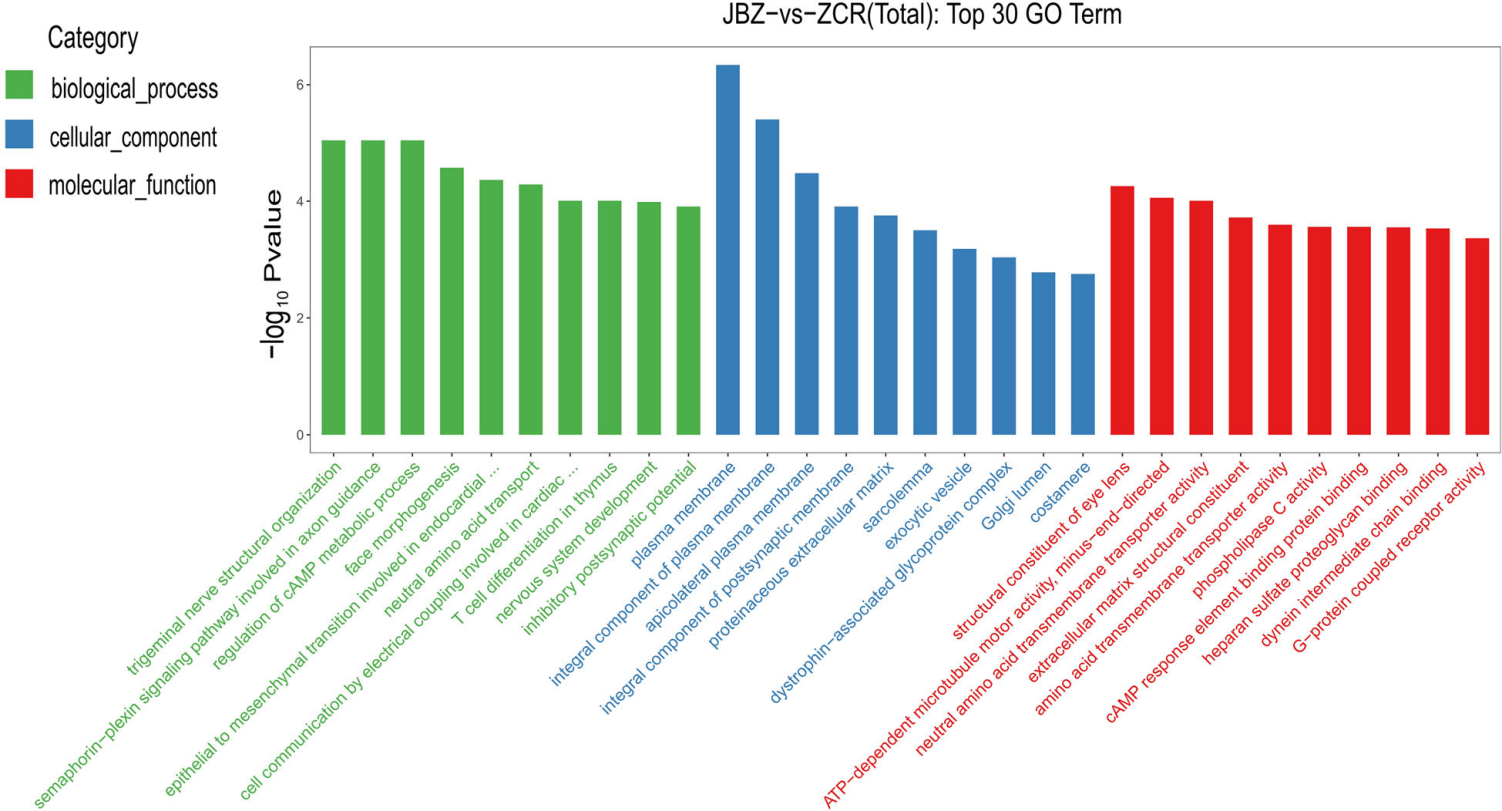
GO enrichment analysis of differentially expressed genes (DEGs). The GO enrichment analysis of the top 30 GO terms. The X-axis represented the different GO terms, the Y-axis represented -log10Pvalue

GO is used to annotate gene function and standardize the description of gene products according to biological process, cellular component, and molecular function categories. We performed GO enrichment analysis by screening GO terms with more than two DEGs in the three gene product classifications, then sorting based on *P* < 0.05 from the largest to the smallest –log10p value, and selecting the top 10 in each category. A total of 848 DEGs (76.3% of all identified DEGs) were mapped to 3646 GO terms, and the top 30 GO terms are shown in Fig. 3. Of these, the most significantly enriched in biological process and molecular function categories were trigeminal nerve structural organization (GO: 0021637, *P* = 9.01E-06) and the structural constituent of the eye lens (GO: 0005212, *P* = 5.51E-05), respectively, while the most enriched in the cellular component category were the plasma membrane (GO: 0005886, *P* = 4.65E-07) and the integral component of the plasma membrane (GO: 0005887, *P* = 3.96E-06). These findings confirmed the accuracy of our sequencing results.

### Analysis of the KEGG pathway

**Fig. 4:**
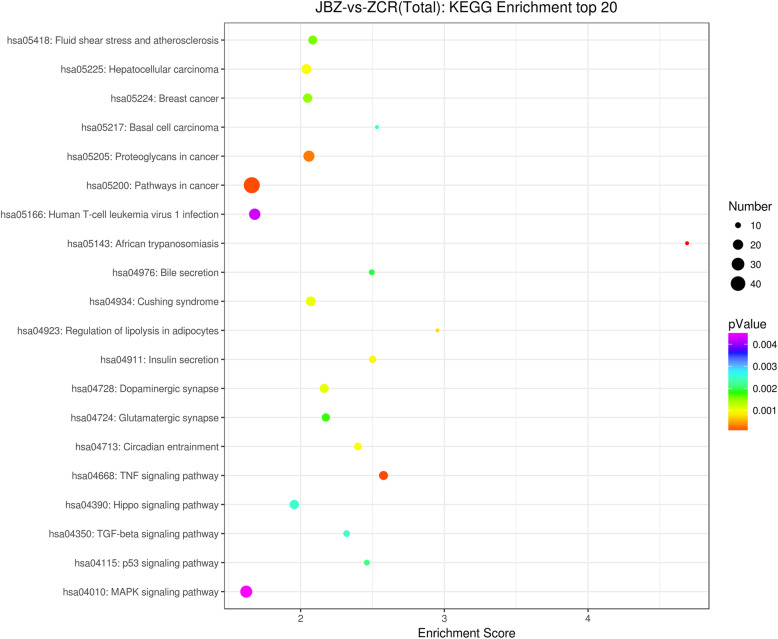
KEGG enrichment analysis of differentially expressed genes (DEGs). As per the citation guidelines (www.kegg.jp/kegg/kegg1.html), KEGG analysis was used to predict the signaling pathways in which these DEGs may be involved [9], KEGG software from the Kanehisa laboratory was used with prior permiossion. The KEGG enrichment analysis of the top 20 was shown in the figure. The number of genes was represented by the size of the circle, and the *P*-value was represented by the color

KEGG is a database for the pathway analysis of DEGs to understand the biological functions of identified genes. In this study, KEGG was divided into the following six classifications: Cellular processing (four categories), Environmental information processing (three categories), Genetic information processing (four categories), Human diseases (eight categories), Metabolism (11 categories), and Organismal systems (10 categories). A total of 409 DEGs (36.8% of all identified DEGs) were assigned to 286 KEGG pathways. The top 20 KEGG enrichment pathways are shown in Fig. 4, and include transforming growth factor (TGF)-β, p53, mitogen-activated protein kinase (MAPK), tumor necrosis factor, and other common signaling pathways. A cancer-related pathway had the highest number of DEGs, suggesting that it might be associated with cell proliferation and apoptosis. KEGG analysis indicated that ARC involves multiple processes and complex signaling pathways, which require further exploration.

### RT-qPCR validation

**Fig. 5:**
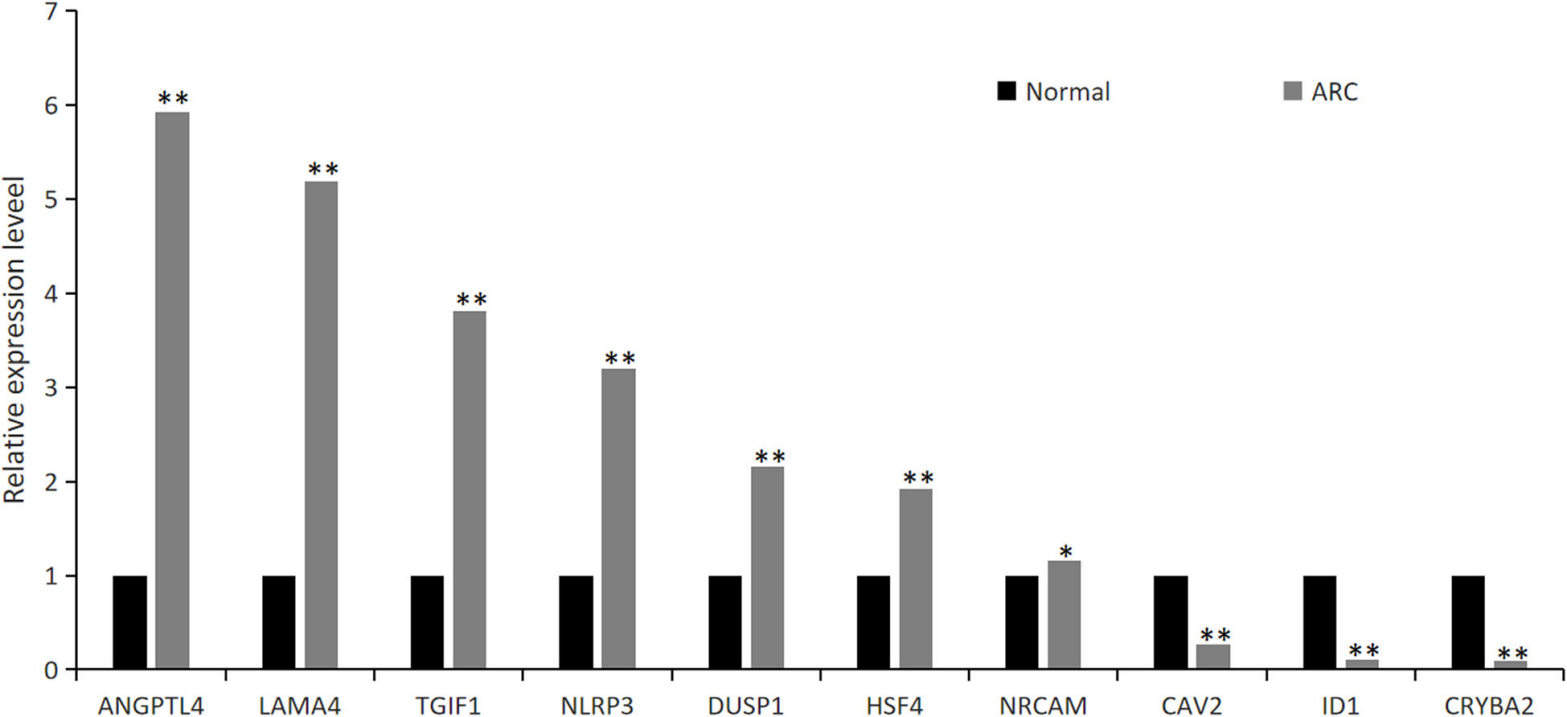
qRT-PCR validation of ten differentially expressed genes. The relative expression levels of genes in ARC patients and normal samples. (*p < 0.05; **P < 0.01)

To confirm RNA sequencing results, 10 DEG transcripts were selected for validation using RT-qPCR. DEG expression levels in the ARC group compared with the control group were consistent with RNA sequencing data, indicating the accuracy of our findings (Fig. 5).

## Discussion

ARC is the leading cause of blindness worldwide, and its incidence is increasing annually. Till date, ARC pathogenesis has mainly been studied at the molecular and cellular levels, with particular focus on the epigenetic changes occurring for multiple genes [11]. Although many genes associated with ARC have been identified, several are undiscovered. In the present study, we conducted in-depth research into ARC-related genes using the bioinformatics technology of transcriptome sequencing.

Here, our study showed that the clean ratio in both groups exceeds 89%, and the highest mapping ratio is 93.5%. In all samples, we identified a total of 17,348 genes, including 1112 genes whose expression level was 2 times or more different than that of the transparent lens. A comparative transcriptome analysis between normal and ARC samples showed that 698 DEGs in ARC were up-regulated and 414 DEGs were down-regulated. These data can provide necessary tissue-specific insights for the ARC transcriptome, such as the number, the expression level, and the distribution of DEGs. It can be used to examine the expression of target genes in ARC, which helps explain genetic-related research. Besides, it can also be used to select candidate genes that are more functionally related to ARC development.

As the only transparent, avascular, and nerveless organ in the human body, the lens is composed of three parts: the lens capsule, lens epithelial cells, and lens fibers [12]. Lens epithelial cells are the only cells that contain a nucleus capable of aerobic metabolism and are closely connected to the anterior lens capsule. Because the stability of lens epithelial cells is important for maintaining lens transparency and metabolic homeostasis [13], we used these cells for sequencing analysis. We obtained a clean reads ratio in both ARC patients and controls of more than 89%, with a highest mapping ratio of 93.5%. From all samples, we identified a total of 17,348 genes, including 1112 whose expression differed by more than 2-fold than that of the transparent lens. Comparative transcriptome analysis between ARC and control samples showed that 698 DEGs were up-regulated and 414 were down-regulated in ARC. These data provide important tissue-specific insights for the ARC transcriptome, and can be used to examine the expression of target genes that are more functionally related to ARC development.

GO term analysis showed that structural constituents of the eye lens were highly enriched in the present study, which undoubtedly verifies the reliability of our results. DEGs in this GO term included *BFSP1*, *LIM2*, *CRYBA4*, *CRYBA2*, *MIP*, *TMOD1*, *GJA1*, *LIM2*, *HSF4*, and *WNT2B*, several of which are closely associated with cataract development. Additionally, some genes that we identified were previously reported to be related to the pathogenesis of ARC, such as *CRYA2* and *HSF4* [14, 15], and we have also verified these genes used RT-qPCR that are consistent with the results. Our transcriptome results also allowed us to identify and verify some genes rarely associated with ARC. Yan et al. found that the increased expression of laminin α4 in the anterior lens capsule is the main mechanism leading to the occurrence of ARC [16], which is consistent with our transcriptome sequencing results. Moreover, the inhibition of *NLRP3* was shown to reduce oxidative stress-induced damage to human lens epithelial cells, while NLRP3 protein expression was significantly increased in lens epithelial cells under oxidative stress [17], which was also consistent with our verification results. However, more than 80% of the genes identified by transcriptome sequencing in this study have not previously been associated with ARC, so should be explored in future in-depth studies.

Some genes of interest identified here could be studied as future candidate genes of ARC. As a member of the cell adhesion molecule (CAM) family, the *NrCAM* gene plays a key role in the development of the nervous system [18, 19]. *NrCAM* expression was reported to be essential for maintaining contact between lens cells, and a lack of *NrCAM* caused lens fiber disorder which ultimately led to the formation of mouse cataracts [20]. However, *NrCAM* expression changes have not been investigated in ARC. We showed by transcriptome sequencing that *NrCAM* was down-regulated in ARC patients compared with controls, but RT-qPCR found it to be elevated in patients, which we speculate might reflect its low expression level. With this in mind, we aim to explore its function in ARC in future studies. We also identified potential ARC candidate genes previously shown to be involved in the occurrence and development of a variety of eye diseases, but not ARC, such as *ANGPTL4*. Aberrant *ANGPTL4* expression levels are associated with branch retinal vein occlusion, macular edema, and age-related macular degeneration diseases, and are considered to be a biomarker and therapeutic target for retinopathy caused by ischemia [21, 22]. However, no studies have directly linked *ANGPTL4* with ARC, so we will investigate this in our future research.

The apoptosis of lens epithelial cells is a common cellular basis for the occurrence and development of ARC [23]. A number of common apoptosis pathways such as p53, MAPK, and TGF-β signaling pathways are closely related to the occurrence of ARC, and several appear in our top 20 KEGG pathways [24–26]. Some genes of interest in these pathways include *DUSP1*, *ID1*, and *CAV2*, which all function in multiple signaling pathways [27–29]. We also verified their expression by RT-qPCR and obtained results consistent with transcriptome sequencing analysis.

## Conclusions

The present study aimed to explore the transcription of genes expressed in the human anterior lens capsule of ARC patients to aid future research into the pathogenesis of ARC and novel therapies. We identified 1112 DEGs between ARC patients and controls using RNA sequencing technology of which many were novel as reported from this study. Although the functions of most identified DEGs have not been explored in ARC, we selected several potential ARC candidate genes for future studies.

## Data Availability

All data files are available from the NCBI database (accession number PRJNA678236), https://www.ncbi.nlm.nih.gov/Traces/study/?acc=PRJNA678236.

## Abbreviations

ARC: Age-related cataract
GO: Gene Ontology
KEGG: Kyoto Encyclopedia of Genes and Genomes
DEGs: Differentially expressed genes
RT-qPCR: Reverse transcription-quantitative polymerase chain reaction
CAMs: Cell adhesion molecules
FC: Fold change
BRVO: Branch retinal vein occlusion
ME: Macular edema
AMD: Age-related macular degeneration

## References

[1] Yao L, Yang L, Song H (2020). MicroRNA miR-29c-3p modulates FOS expression to repress EMT and cell proliferation while induces apoptosis in TGF-beta2-treated lens epithelial cells regulated by lncRNA KCNQ1OT1. Biomed Pharmacother.

[2] Knopfel EB, Vilches C, Camargo SMR (2019). Dysfunctional LAT2 amino acid transporter is associated with cataract in mouse and humans. Front Physiol.

[3] Ono K, Hiratsuka Y, Murakami A (2010). Global inequality in eye health: country-level analysis from the global burden of disease study. Am J Public Health.

[4] Wang Z, Su D, Sun Z, Liu S, sun L, Li Q, et al. MDM2 phosphorylation mediates H2O2-induced lens epithelial cells apoptosis and age-related cataract. Biochem Biophys Res Commun. 2020;528(1):112–9. 10.1016/j.bbrc.2020.05.060. 10.1016/j.bbrc.2020.05.060 32471716

[5] Asbell PA, Dualan I, Mindel J (2005). Age-related cataract. Lancet.

[6] Hawse JR, Hejtmancik JF, Huang QL, et al. Identification and functional clustering of global gene expression differences between human age-related cataract and clear lenses. Mol Vis. 2003;9:515–37. PMC2831407 14551530

[7] Hejtmancik JF, Kantorow M (2004). Molecular genetics of age-related cataract. Exp Eye Res.

[8] Kim D, Langmead B, Salzberg SL (2015). HISAT: a fast spliced aligner with low memory requirements. Nat Methods.

[9] Kanehisa M, Goto S (2000). KEGG: Kyoto encyclopedia of genes and genomes. Nucleic Acids Res.

[10] Livak KJ, Schmittgen TD (2001). Analysis of relative gene expression data using real-time quantitative PCR and the 2(−Delta Delta C(T)) method. Methods..

[11] Kantorow M, Kays T, Horwitz J (1998). Differential display detects altered gene expression between cataractous and normal human lenses. Invest Ophthalmol Vis Sci.

[12] Vendra VPR, Khan I, Chandani S (2016). Gamma crystallins of the human eye lens. Biochimica et Biophysica Acta.

[13] Ghosh KS, Chauhan P (2019). Crystallins and their complexes. Subcell Biochem.

[14] Puk O, Ahmad N, Wagner S, Hrabé de Angelis M, Graw J (2011). First mutation in the betaA2-crystallin encoding gene is associated with small lenses and age-related cataracts. Invest Ophthalmol Vis Sci.

[15] Shi Y, Shi X, Jin Y (2008). Mutation screening of HSF4 in 150 age-related cataract patients. Mol Vis.

[16] Yan Y, Yu HY, Sun LY, Liu H, Wang C, Wei X, et al. Laminin α4 overexpression in the anterior lens capsule may contribute to the senescence of human lens epithelial cells in age related cataract. Aging. 2019;11(9):2699–723. 10.18632/aging.101943. 10.18632/aging.101943 PMC6535067 31076560

[17] Zou YY, Cui BJ, Liang P, Tian X, Ma Y, Zhao S (2020). Inhibition of NLRP3 protects human Lens epithelial cells against oxidative stress-induced apoptosis by NF-kappaB signaling. Ophthalmic Res.

[18] Grurnet M, Mauro V, Burgoon MP (1991). Structure of a new nervous system glycoprotein, nr-CAM, and its relationship to subgroups of neural cell adhesion molecules. J Cell Biol.

[19] Kayyem JF, Roman JM, de la Rosa Ei J (1992). Bravo/nr-CAM is closely related to the cell adhesion molecules L1 and ng-CAM and has a similar heterodimer structure. The Journal of CeU Biolog.

[20] More MI, Kirsch FP, Rathjen FG (2001). Targeted ablation of NrCAM or ankyrin-B results in disorganized lens fibers leading to cataract formation. J Cell Biol.

[21] Kim JH, Shin JP, Kim IT (2016). Aqueous Angiopoietin-like 4 levels correlate with nonperfusion area and macular edema in branch retinal vein occlusion. Invest Ophthalmol Vis Sci.

[22] Kim JH, Shin JP, Kim IT, Park DH (2018). Angiopoietin-like 4 correlates with response to intravitreal ranibizumab injections in neovascular age-related macular degeneration. Retina..

[23] Li WC, Kuszak JR, Dunn K, Wang RR, Ma W, Wang GM, et al. Lens epithelial cell apoptosis appears to be a common cellular basis for non-congenital cataract development in humans and animals. J Cell Biol. 1995;130(1):169–81. 10.1083/jcb.130.1.169. 10.1083/jcb.130.1.169 PMC2120521 7790371

[24] Qin Y, Zhao JY, Min XJ (2014). MicroRNA-125b inhibits lens epithelial cell apoptosis by targeting p53 in age-related cataract. Biochimica et Biophysica Acta.

[25] Du SS, Shao JZ, Xie DD (2020). Decorin inhibits glucose-induced lens epithelial cell apoptosis via suppressing p22phox-p38 MAPK signaling pathway. PLoS One.

[26] Hales AM, Chamberlain CG, McAvoy JW (2000). Susceptibility to tgfb2-induced cataract increases with aging in the rat. Invest Ophthalmol Vis Sci.

[27] Yang JX, Sun LG, Han J, Zheng W, Peng W (2019). DUSP1/MKP-1 regulates proliferation and apoptosis in keratinocytes through the ERK/Elk-1/Egr-1 signaling pathway. Life Sci.

[28] Huang YH, Hu J, Chen F, Lecomte N, Basnet H, David CJ, et al. ID1 mediates escape from TGFbeta tumor suppression in pancreatic Cancer. Cancer Discov. 2020;10(1):142–57. 10.1158/2159-8290.CD-19-0529. 10.1158/2159-8290.CD-19-0529 PMC6954299 31582374

[29] Liu F, Shangli Z, Hu ZL (2018). CAV2 promotes the growth of renal cell carcinoma through the EGFR/PI3K/Akt pathway. Onco Targets Ther.

